# Diffusion Tensor Imaging of Skeletal Muscle Contraction Using Oscillating Gradient Spin Echo

**DOI:** 10.3389/fneur.2021.608549

**Published:** 2021-02-15

**Authors:** Valentina Mazzoli, Kevin Moulin, Feliks Kogan, Brian A. Hargreaves, Garry E. Gold

**Affiliations:** Department of Radiology, Stanford University, Stanford, CA, United States

**Keywords:** MRI, DTI, OGSE, diffusion MRI, oscillating gradients, muscle contraction

## Abstract

Diffusion tensor imaging (DTI) measures water diffusion in skeletal muscle tissue and allows for muscle assessment in a broad range of neuromuscular diseases. However, current DTI measurements, typically performed using pulsed gradient spin echo (PGSE) diffusion encoding, are limited to the assessment of non-contracted musculature, therefore providing limited insight into muscle contraction mechanisms and contraction abnormalities. In this study, we propose the use of an oscillating gradient spin echo (OGSE) diffusion encoding strategy for DTI measurements to mitigate the effect of signal voids in contracted muscle and to obtain reliable diffusivity values. Two OGSE sequences with encoding frequencies of 25 and 50 Hz were tested in the lower leg of five healthy volunteers with relaxed musculature and during active dorsiflexion and plantarflexion, and compared with a conventional PGSE approach. A significant reduction of areas of signal voids using OGSE compared with PGSE was observed in the tibialis anterior for the scans obtained in active dorsiflexion and in the soleus during active plantarflexion. The use of PGSE sequences led to unrealistically elevated axial diffusivity values in the tibialis anterior during dorsiflexion and in the soleus during plantarflexion, while the corresponding values obtained using the OGSE sequences were significantly reduced. Similar findings were seen for radial diffusivity, with significantly higher diffusivity measured in plantarflexion in the soleus muscle using the PGSE sequence. Our preliminary results indicate that DTI with OGSE diffusion encoding is feasible in human musculature and allows to quantitatively assess diffusion properties in actively contracting skeletal muscle. OGSE holds great potential to assess microstructural changes occurring in the skeletal muscle during contraction, and for non-invasive assessment of contraction abnormalities in patients with muscle diseases.

## Introduction

Diffusion tensor imaging (DTI) is an MRI-based technique that allows to measure the anisotropic diffusion of water molecules in muscle tissue. DTI can non-invasively provide *in vivo* information on tissue architecture and microstructure, either normal or in a diseased state. In a typical DTI experiment, diffusion is probed along multiple directions using diffusion encoding gradients. The application of diffusion encoding gradients results in a signal attenuation, which across several directions can be geometrically interpreted by a rank 2 tensor. This tensor can be diagonalized to derive the principal directions of diffusion. In skeletal muscle, the highest diffusivity is observed along the axis of the fiber [axial diffusivity (AD)] and the lowest in the fiber cross section [radial diffusivity (RD)]. Due to its exquisite sensitivity to tissue microstructure, DTI is becoming an increasing popular tool to assess skeletal muscle status in a wide range of diseases (1) and muscle injuries (2).

Muscle contraction involves muscle fiber shortening and increase in cross-sectional area (CSA). The ability of accurately measuring these structural changes during muscle contraction *in vivo* has the potential to elucidate contraction abnormalities that are not apparent in relaxed musculature. Extensive simulation work showed the high sensitivity of skeletal muscle DTI to cellular size (3), with higher diffusion coefficients and lower fractional anisotropy (FA) associated with increasing fiber CSA. Due to its high cellular size sensitivity, DTI is a very promising tool to assess *in vivo* and non-invasively skeletal muscle microstructure and microstructural changes due to contraction in human musculature. Previous work has shown the high sensitivity of DTI to transient structural changes induced by passive shortening and lengthening (4–6) with increased radial diffusivity associated with passive shortening and increase in the fascicle CSA. Active muscle contraction induces shortening of muscle fibers, associated with increased fiber CSA. DTI is therefore an attractive tool to non-invasively assess muscle contraction mechanism by measuring changes in radial diffusivity during active contraction with respect to relaxed musculature.

DTI has been extensively applied to probe skeletal muscle microstructure in healthy subjects and patients. However, most of this work has been performed for static, non-contracting muscle, mostly due to the presence of signal voids in diffusion-weighted images acquired during muscle contraction (7, 8). These areas of signal void are caused by incoherent motion within the contracting muscle tissue (9), which results in rapid signal dephasing in contracting muscle and preclude further analyses and extraction of microstructural information from the DTI measurement. This need for static acquisition largely prohibits the characterization of fundamental functional aspects of the skeletal muscle, such as microstructural changes due to contraction and contraction-induced diffusion response. Recent work has shown that the presence and extent of signal voids occurring during external muscle stimulation in diffusion MRI can provide information on motor units (10). However, these methods do not allow to characterize the microstructural features of skeletal muscle.

Diffusion behavior in skeletal muscle has long been shown to be highly time-dependent (11), with diffusion coefficients measured using DTI that strongly depends on the time allowed for diffusing water molecules to probe the local environment (the so-called “diffusion dime”). This time-dependent behavior has also been combined with advanced mathematical modeling to derive cell size (12). For increasing diffusion times, the water molecules will interact with more barriers, and the apparent diffusion coefficient (ADC) will decrease, eventually reaching an asymptotic lower value (13, 14). Skeletal muscle is a highly hierarchical structure, and it is therefore possible to study different levels of tissue organization by tuning the diffusion time accordingly.

DTI in skeletal muscle is typically performed using pulsed gradient spin echo (PGSE) diffusion encoding, which has an intermediate diffusion time (about 20-30 ms), resulting in an inability to provide information at the level of the individual muscle fibers, typically on the order of 30–60μm. In practice, PGSE is also extremely sensitive to bulk motion caused by voluntary or involuntary muscle contraction, which leads to unwanted signal dropouts in the acquired images. Diffusion methods with very long diffusion times such as stimulated echoes are also routinely used to investigate muscle fiber size (15) but suffer from the same issue as the PGSE approach. OGSE has been proposed in the brain imaging field as an efficient method to reduce the diffusion time of the experiment while maintaining a sufficient amount of diffusion encoding (16, 17). By design, cosine or trapezoid-cosine OGSE waveforms also provide full motion compensation (M_0_ = M_1_ = M_2_ = 0), which could be particularly useful to compensate for bulk tissue motion induced by muscle contraction. Additionally, its short diffusion times could allow to investigate ultrastructural features that are currently not accesible using diffusion encoding schemes with longer diffusion times. OGSE could also allow to study microstructural and ultrastructural changes in actively contracted muscles. The OGSE method, although promising, is mostly confined to neurological (16, 18) and cancer (19) application and has never been applied to the skeletal muscle to date.

Therefore, the aim of this work is to explore OGSE for evaluation of the human skeletal muscle on a clinical 3T MRI scanner and to exploit its inherent motion compensation to measure muscle microstructure during active muscle contraction.

## Methods

### Subjects

Five healthy volunteers (four females and one male) were recruited for lower-leg MRI imaging. None of the subjects had a history of muscle disease, and they were asked to refrain from any strenuous physical activities the day before the scan. This study was approved by the University Institutional Review Board. We received informed consent from all subjects prior to the study, according to our institution's regulations.

### Data Acquisition

**Figure 1:**
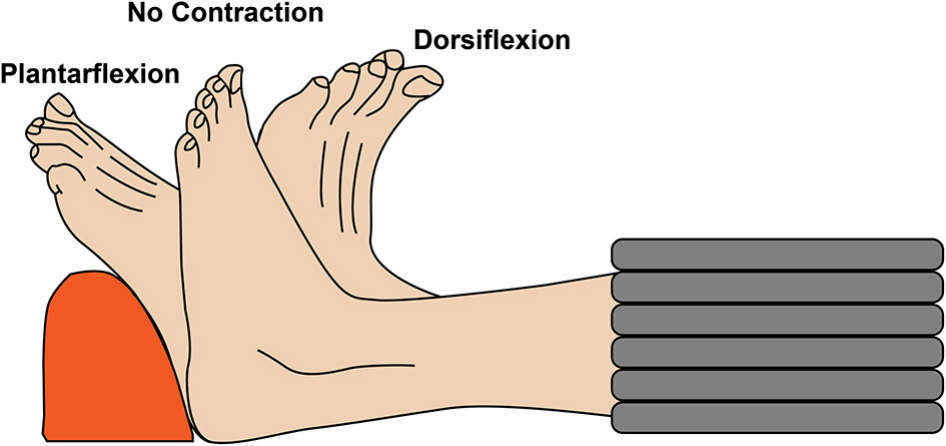
Schematic representation of the experimental setup. The left lower leg of the volunteers was scanned with relaxed musculature and during active foot dorsiflexion and plantarflexion.

**Figure 2:**
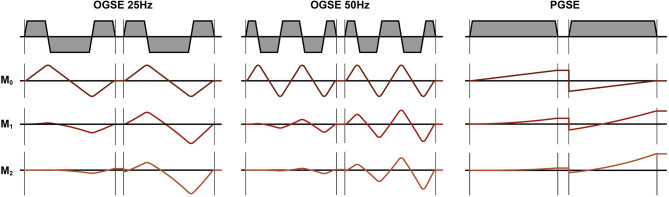
Diffusion encoding gradients for oscillating gradient spin echo (OGSE) 25 Hz, OGSE 50 Hz, and pulsed gradient spin echo (PGSE). The OGSE waveforms are motion compensated (M_0_ = M_1_ = M_2_ = 0), while the PGSE is not motion compensated (M_0_ = 0, M_1_ ≠ 0, M_2_ ≠ 0).

MR imaging was performed with a 3T MRI scanner (SIGNA 750w Premier, GE Healthcare) with maximal nominal gradient strength = 80 mT/m and max nominal slew rate = 120 mT/m/s. A receive-only medium-size 16-channel flexible coil array (NeoCoil, Pewaukee, WI, USA) wrapped around the left calf was used for signal reception. All subjects were placed supine, feet first in the scanner, with both legs in the full extended position. The scan protocol consisted of a series of DTI scans acquired with relaxed musculature and during foot dorsiflexion and plantarflexion. Sandbags on both sides of the left leg were used to minimize displacement of the subject between acquisitions. DTI scans were performed in three configurations: (1) at rest, (2) with the subjects actively contracting their muscle to maintain a plantarflexion position, while pushing their foot against a rigid support, and (3) with the subject actively contracting their muscles to achieve the maximum level of dorsiflexion (Figure 1). Three different DTI sequences (PGSE, OGSE 25 Hz and OGSE 50 Hz) were collected for each position (no contraction, dorsiflexion, and plantarflexion), resulting in nine DTI acquisitions per subject. The order of positions and DTI acquisitions was randomized to reduce the effect of perfusion and muscle fatigues on the DTI results. For the OGSE acquisition, cosine trapezoidal gradient waveforms (20) with 50 and 25 Hz, corresponding to *N* = 2 and *N* = 1 oscillations, were implemented. This resulted in a diffusion time of 7.5 and 4.1 ms for the OGSE 25 Hz and OGSE 50 Hz sequences, respectively (16). For both OGSE frequencies, the encoding waveform duration before and after the refocusing pulse was 40 ms long (Figure 2). The PGSE sequence was designed to match the timing of the OGSE sequence and was thus composed of a single monopolar trapezoid gradient with 40-ms duration. For each DTI acquisition, the maximum gradient strength used was adjusted in order to achieve the same maximum b-value of 180 s/mm^2^. For the three DTI acquisitions (PGSE, OGSE 25 Hz, and OGSE 50 Hz), diffusion was encoded along 15 non-collinear diffusion encoding directions, and three non-diffusion-weighted volumes were acquired. Two averages were acquired for the diffusion-weighted volume, for a total of 33 scanned volumes per sequence. Other common scan parameters were repetition time/echo time (TR/TE) = 2,800/94 ms, field of view (FOV) = 160 × 160 mm^2^, 10 slices, voxel size = 2.7 × 2.7 × 10 mm^3^. Spectral spatial water excitation was used for fat suppression. The total scan time for each DTI dataset was 1 min 30 s.

### Image Processing

In order to estimate the signal-to-noise ratio (SNR), we calculated the standard deviation of the noise (σ) from two identically acquired non-diffusion-weighted and diffusion-weighted images (*b* = 0 mm^2^/s and *b* = 180 mm^2^/s) for all the scans acquired in neutral position. SNR was defined as the mean of the signal over all muscles in the lower leg divided by σ. All images were visually inspected, and the areas of signal voids in the anterior and posterior compartments of the lower leg in each DTI scan were counted in a mid-calf slice (Slice 4) in 30 images (15 diffusion encoding directions ^*^ 2 averages). Areas of signal voids were classified as area of four or more contiguous black pixels in the muscle of interest.

All DTI scans were denoised using a principal component analysis (PCA) denoising algorithm (21). The non-diffusion-weighted scan acquired using the PGSE sequence with the lower leg in relaxed position was used as an anatomical reference. For each subject, all other DTI datasets were non-rigidly registered to the reference scan, in order to take into account the change in shape of the muscle due to active contraction. All image registrations were performed using Elastix (22), with a b-spline algorithm with the metric Advanced Mattes Mutual information = and a b-spline interpolation order of three. The number of resolutions for the registration was set to two. All image registrations was performed using Elastix (22). The reference image was also used to manually delineate the tibialis anterior and soleus muscles. After registration, the diffusion images per acquisition were fitted into a DTI model using a WLLS algorithm (23) with outlier rejections (24). The first eigenvalue of the DTI tensor correspond to the AD (= λ_1_) and the mean of the second and third eigenvalues to the RD [=(λ_2_ + λ_3_)/2]. All image processing and tensor fitting was performed using QMRITools (25) (https://mfroeling.github.io/QMRITools).

### Statistical Analysis

Differences in number of signal voids, AD, RD, and FA, were tested using a mixed-model ANOVA, accounting for subject, DTI sequence (PGSE, OGSE 25 Hz, and OGSE 50 Hz), and leg position (no contraction, active dorsiflexion, and active plantarflexion). *Post-hoc* Tukey test was used to examine individual relationships. All statistical analyses were performed using SPSS. Additionally, a one-way ANOVA with a Fisher correction was used to test the effect of contraction status for the diffusion parameters obtained with OGSE 25 Hz and OGSE 50 Hz sequences. All tests were performed two-sided with significance set at *p* < 0.05.

## Results

All results were visually inspected and were considered of sufficient quality to continue with further analyses. The mean SNR for the non-diffusion-weighted scan was 32 ± 2, 28 ± 2, and 28 ± 2 for OGSE 25 Hz, OGSE 50 Hz, and PGSE, respectively. The mean SNR for the diffusion-weighted scans was 16 ± 4, 16 ± 1, and 17 ± 1 for OGSE 25 Hz, OGSE 50 Hz, and PGSE, respectively.

**Figure 3:**
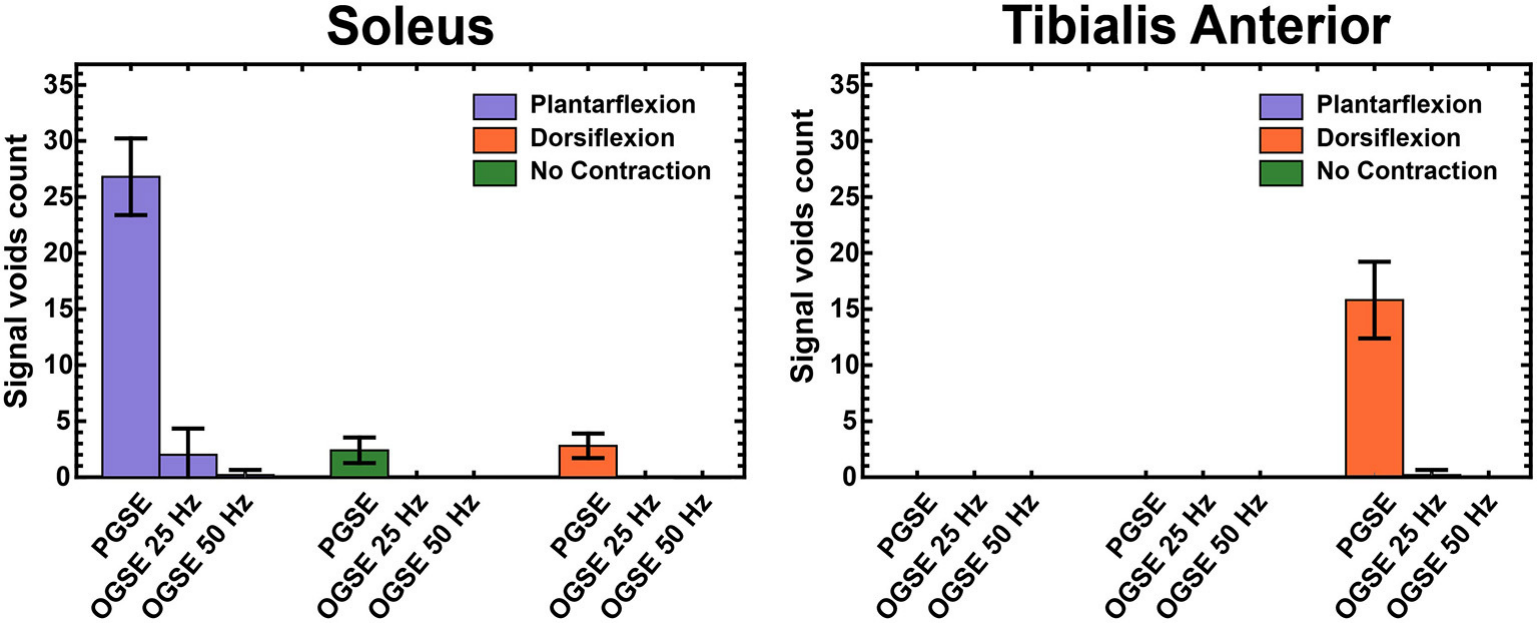
Counts of areas with extended signal voids in Slice 4 (mid-calf) in the soleus and tibialis anterior muscles, averaged over all volunteers.

Figure 3 summarizes the results of visual counting of areas of extended signal voids in diffusion-weighted volumes at different contractions and using different diffusion encoding waveforms. For the images acquired with relaxed musculature, areas of signal voids were present for each volunteer using the PGSE sequence. These areas of signal voids, likely originating by involuntary muscle twitch (7), were localized in the posterior compartment of the leg and were not present when encoding diffusion using OGSE waveforms. No signal voids were observed in relaxed musculature in the tibialis anterior muscle with any of the waveforms. Images acquired using PGSE diffusion encoding during active plantarflexion had significantly more areas of signal voids in the soleus than using OGSE 25 Hz and OGSE 50 Hz (*p* < 0.0001). These artifacts were present in every subject. OGSE waveforms resulted in a significantly reduced number of signal voids (observed in three subjects and one subject for the OGSE 25 Hz and OGSE 50 Hz, respectively). Similarly, areas of signal voids were observed in the tibialis anterior muscle for every volunteer during active dorsiflexion but were significantly reduced when using OGSE (*p* < 0.0001 for both OGSE 25 Hz and OGSE 50 Hz) for the same type of contraction.

**Figure 4:**
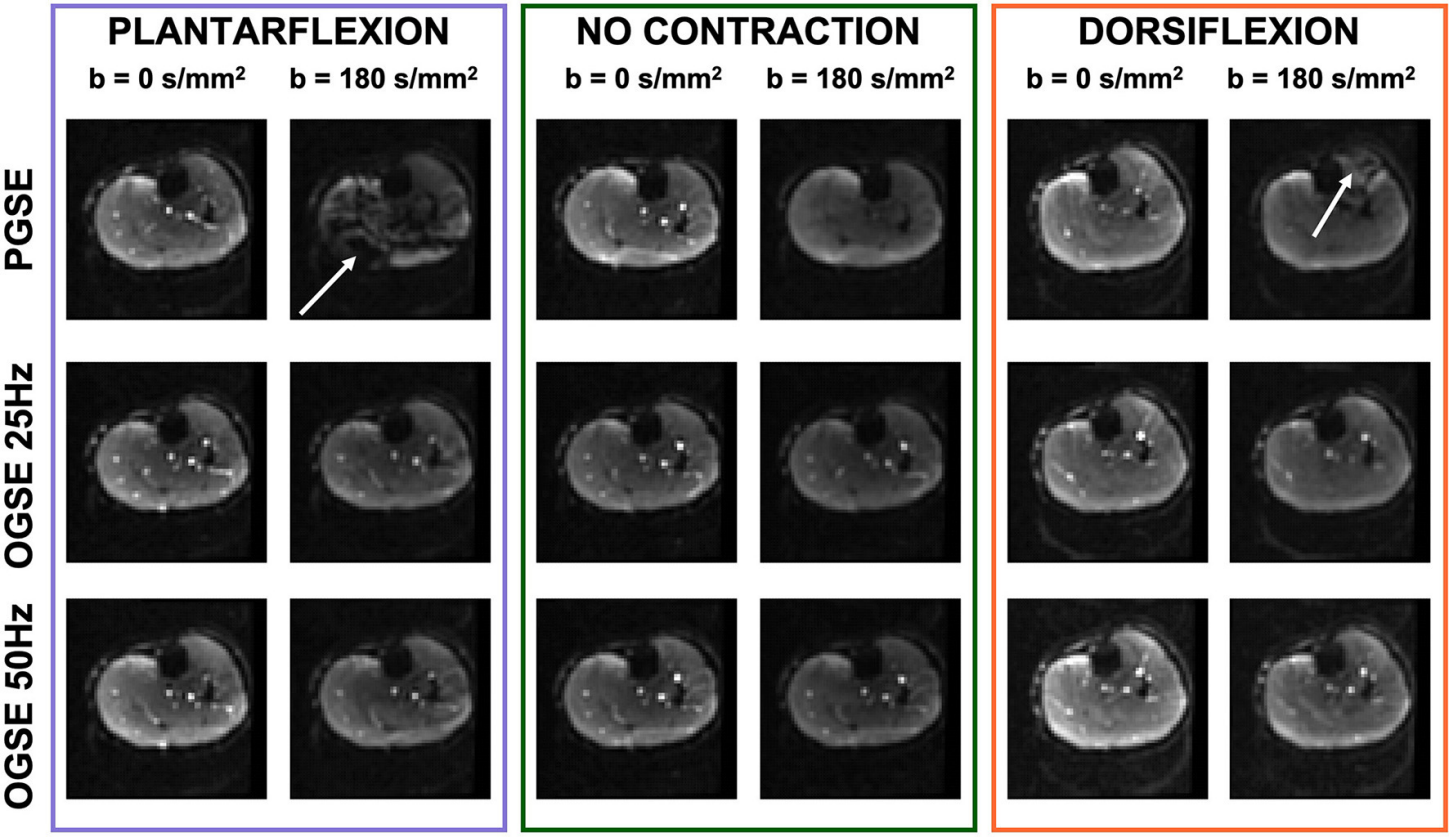
Non-diffusion-weighted and diffusion-weighted scans of one volunteer acquired during active plantarflexion (purple), with relaxed musculature (no contraction, green), and during active dorsiflexion (orange). The pulsed gradient spin echo (PGSE) scans show localized areas of signal voids (white arrow) originating by spin dephasing during muscle activation. Note the bright signal from blood vessels in the diffusion-weighted scans obtained with oscillating gradient spin echo (OGSE), indicating motion compensation.

Representative images of the calf of a female volunteer showing images acquired using OGSE 25 Hz, OGSE 50 Hz, and PGSE are shown in Figure 4. PGSE scans acquired during active muscle contraction showed clear areas of signal voids localized in the anterior compartment of the lower leg (for the dorsiflexed foot position) and in the posterior compartment (for the plantarflexed foot position). The presence of these areas was dramatically reduced in the scans acquired using OGSE 25 Hz and OGSE 50 Hz, with either relaxed or contracted musculature.

**Figure 5:**
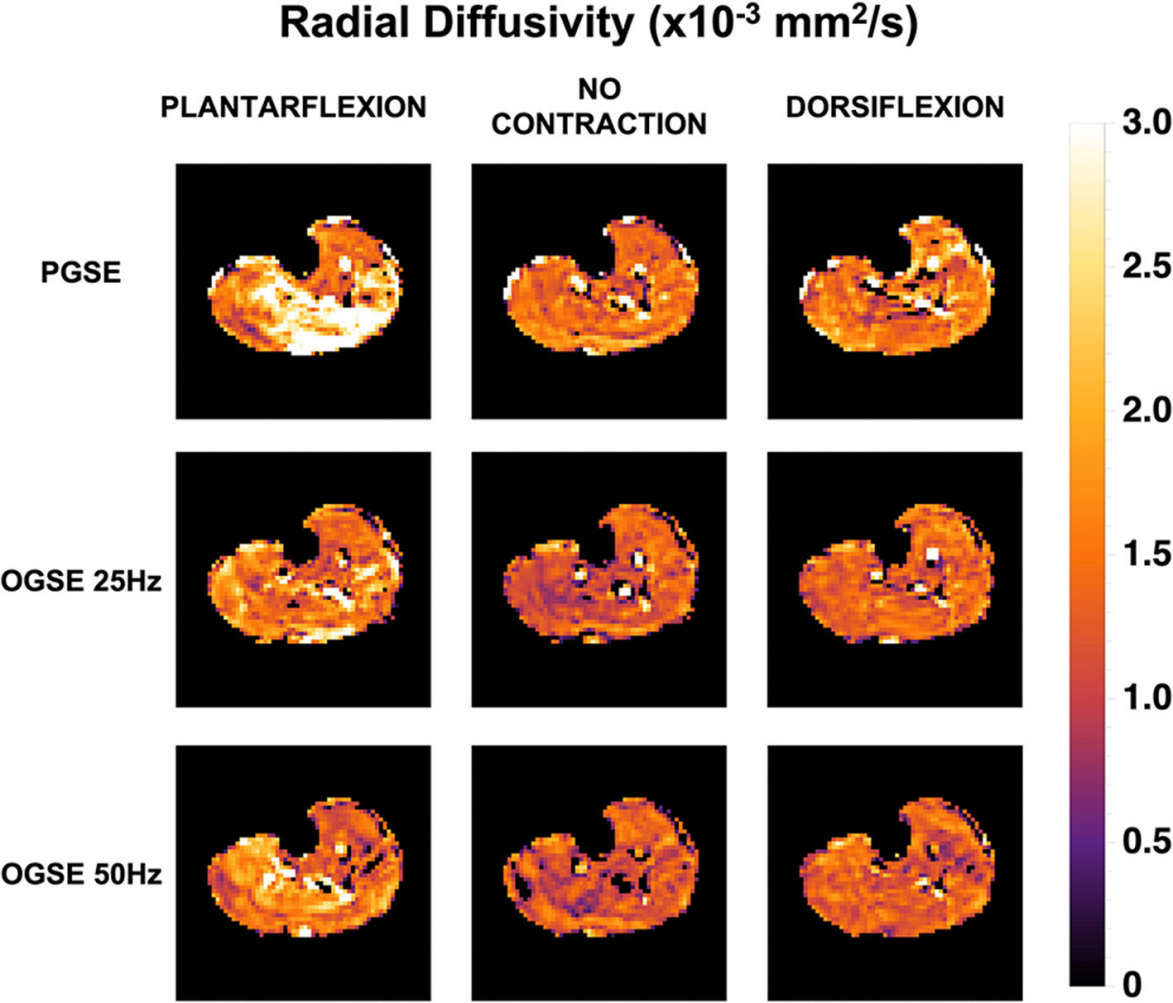
Radial diffusivity (RD) maps for one female volunteer. Abnormally elevated diffusion values are observed in the posterior compartment of the lower leg during plantarflexion and in the anterior compartment during dorsiflexion when using a pulsed gradient spin echo (PGSE) sequence.

**Figure 6:**
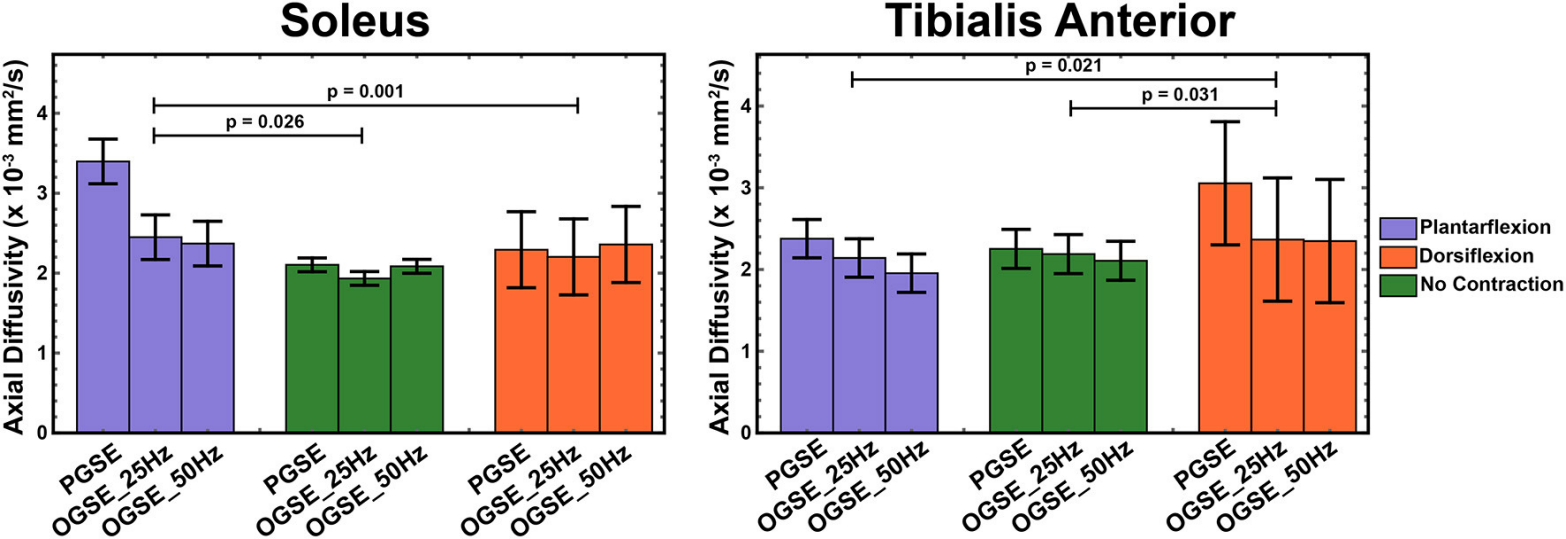
Axial diffusivity (AD) measured during active plantarflexion (purple), with relaxed musculature (green), and during active dorsiflexion (orange) using three different diffusion encoding sequences [pulsed gradient spin echo (PGSE), oscillating gradient spin echo (OGSE) 25 Hz, and OGSE 50 Hz]. Elevated AD values are present in the soleus during active plantarflexion and in the tibialis anterior during active dorsiflexion.

**Figure 7:**
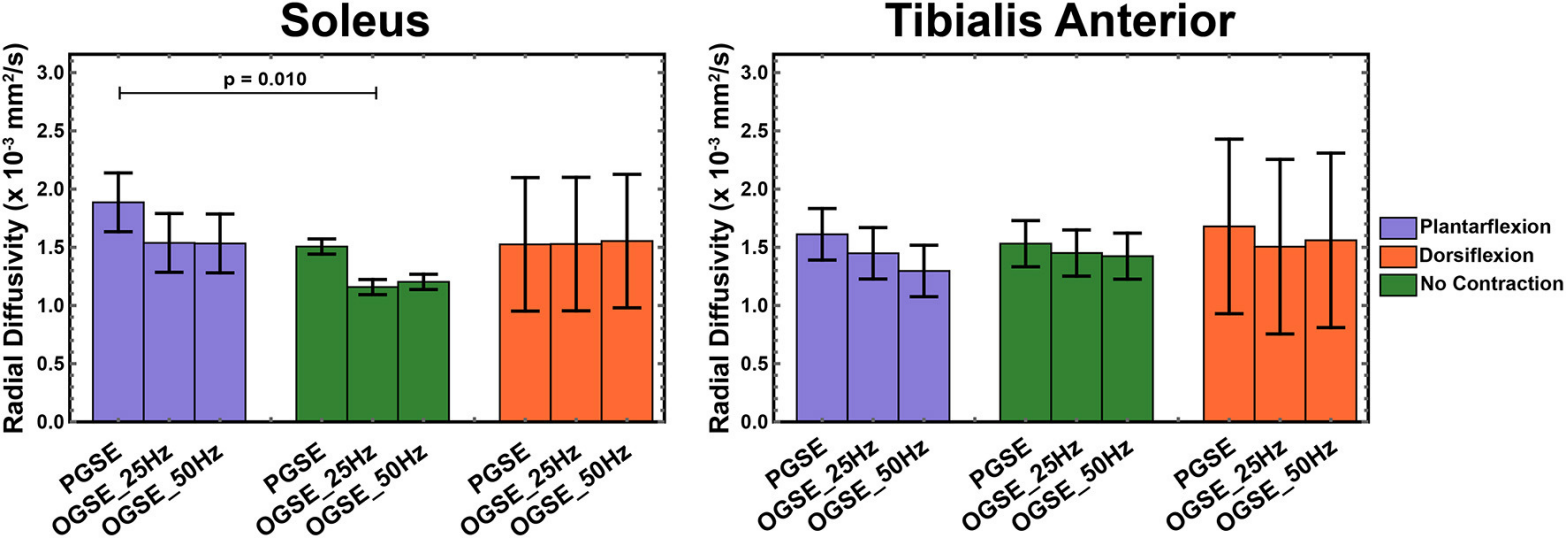
Radial diffusivity (RD) measured during active plantarflexion (purple) and active dorsiflexion (orange) and with relaxed musculature (green) using three different diffusion encoding sequences [pulsed gradient spin echo (PGSE), oscillating gradient spin echo (OGSE) 25 Hz, and OGSE 50 Hz]. Elevated RD values are present in the soleus during active plantarflexion.

Representative RD maps for one volunteer acquired with the three different sequences and three different contraction status are shown in Figure 5. The areas of signal voids present in the diffusion-weighted images acquired with PGSE during active dorsiflexion and plantarflexion resulted in abnormally elevated values of AD and RD in the soleus and tibialis anterior muscles, respectively (Figures 6, 7).

The results of a mixed-model ANOVA showed significantly higher AD values in the tibialis anterior muscle during active dorsiflexion than in plantarflexion (*p* = 0.021) and no-contraction positions (*p* = 0.031). These differences were driven by the abnormally elevated AD values measured in dorsiflexion using PGSE (3.05 ± 0.51 mm^2^/s) compared with OGSE 25 Hz (2.37 ± 0.51 mm^2^/s) and OGSE 50 Hz (2.35 ± 0.75 mm^2^/s). Elevated AD values in the tibialis anterior were observed for the PGSE sequence compared with OGSE 50 Hz (*p* = 0.024). Abnormally elevated AD values were observed in the soleus during plantarflexion using the PGSE sequence (3.40 ± 1.05 mm^2^/s), but these were in a normal range when using the OGSE sequences (2.45 ± 0.41 mm^2^/s for OGSE 25 Hz and 2.37 ± 0.28 mm^2^/s for OGSE 50 Hz). Similar trends of elevated AD were also observed in the soleus when using the PGSE sequence (PGSE vs. OGSE 25 Hz, *p* = 0.053), with higher AD values during plantarflexion compared with dorsiflexion (*p* = 0.026) and no contraction (*p* = 0.001).

The OGSE sequences detected significantly higher AD in the soleus during active plantarflexion compared with no-contraction position (*p* = 0.013), but no significant differences were observed for the other positions (*p* > 0.081). No differences in AD were detected between positions for the tibialis anterior (*p* > 0.117), but a trend of increasing AD in dorsiflexion was observed.

We observed higher RD values in the tibialis anterior muscle when using PGSE during active dorsiflexion (1.68 ± 0.18 mm^2^/s for PGSE vs. 1.52 ± 0.38 mm^2^/s for OGSE 25 Hz and 1.56 ± 0.75 mm^2^/s), although the interaction of sequence and position was not significant (*p* = 0.928). The RD values in the soleus, similarly to AD, were significantly higher in plantarflexion compared with no-contraction position (*p* = 0.010), but no significant difference was detected between plantarflexion and dorsiflexion positions (*p* = 0.579). These differences were primarily driven by the higher RD values observed in plantarflexion using the PGSE sequence (1.88 ± 0.34 mm^2^/s) compared with OGSE 25 Hz (1.54 ± 0.37 mm^2^/s) and OGSE 50 Hz (1.53 ± 0.25 mm^2^/s).

When using an OGSE approach, RD in the soleus during no contraction was significantly lower than in plantarflexion (*p* = 0.028) and dorsiflexion (*p* = 0.026). No differences in RD were observed for the tibialis anterior between different leg positions (*p* > 0.325), although a trend of increasing RD in dorsiflexion was observed.

**Figure 8:**
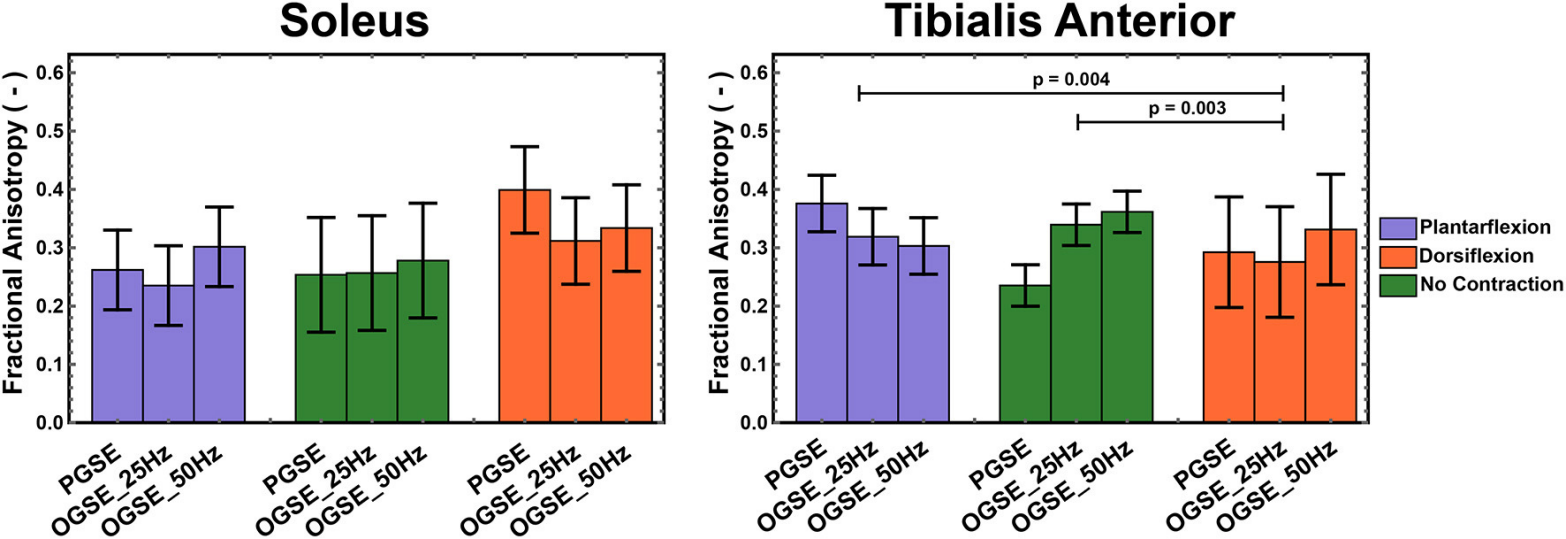
Fractional anisotropy (FA) measured during active plantarflexion (purple) and active dorsiflexion (orange) and with relaxed musculature (green) using three different diffusion encoding sequences [pulsed gradient spin echo (PGSE), oscillating gradient spin echo (OGSE) 25 Hz, and OGSE 50 Hz]. Significantly lower FA values were measured in the tibialis anterior during active foot dorsiflexion.

FA values in the soleus and tibialis anterior muscles, averaged over all volunteers, are reported in Figure 8. In the tibialis anterior, the FA values were significantly lower in dorsiflexion compared with plantarflexion (*p* = 0.004) and no-contraction position (*p* = 0.003). An interaction effect was observed for FA in the soleus (*p* = 0.004), but no separate effect of position (*p* = 0.303) or sequence (*p* = 0.341). No significant differences were observed between OGSE 25 Hz and OGSE 50 Hz sequences for any of the diffusion quantitative parameters (*p* > 0.1).

No significant differences for different foot positions were observed for FA in either soleus (*p* > 0.090) or tibialis anterior (*p* > 0.102) muscles when using OGSE.

## Discussion

This study applied DTI with OGSE diffusion encoding in the musculature of the lower leg to characterize diffusion behavior in actively contracting skeletal muscle for the first time. More than half of the diffusion-weighted volumes acquired using the PGSE sequence during active muscle contraction showed extended areas of signal voids that led to an unrealistic overestimation of diffusion parameters. These artifacts were significantly reduced when using the OGSE sequences and led to physically plausible diffusion values, indicating that trapezoid-cosine OGSE waveforms, due to their motion compensation design, might be promising to investigate muscle microstructure during active muscle contraction.

Areas of signal voids in healthy resting muscle at moderate b-values and associated with a spontaneous incoherent mechanical activity result in local signal dephasing when using stimulated echoes (9) and need to be discarded prior to further analyses and DTI fitting (26). Steidle et al. (9) reported clear and extended areas of signal in resting musculature or the lower leg at b-values as low as 100 s/mm^2^ and a higher occurrence of signal voids in the posterior compartment compared with the anterior compartment, similarly to what was observed in our study. These signal voids, observed in resting musculature, have been associated with incoherent motion of muscle fibers due to a spontaneous muscle activity (7–9). Electrical muscle stimulation of calf muscle has been shown to increase the amount and spatial extent of signal voids in diffusion-weighted scans acquired at low b-values along a single direction, suggesting the sensitivity of diffusion MRI to size and shape of motor units (10, 27). While these results obtained during external stimulation clearly demonstrate the potential of diffusion MRI to study contraction mechanisms, being able to assess muscle microstructure during active contraction would increase the clinical applicability of the method.

The use of PGSE waveforms led to unrealistically and non-physical AD values when performing DTI experiments in actively contracting muscle (soleus during plantarflexion and tibialis anterior during dorsiflexion), while no differences were observed for between the two OGSE sequences. OGSE showed increased RD in the soleus during plantarflexion compared with neutral position, possibly indicating increased muscle CSA due to active contraction, similar to previous findings in passively shortened muscle (4–6, 28, 29). Since increased CSA is expected for increasing applied force, future investigations will focus on the effect of force on measured RD. However, while AD in passively contracted muscles has been shown to be largely unaffected, interestingly in this study, we observed an increase in AD in the soleus during plantarflexion. Muscle contraction involves shortening of the sarcomeres. However, since the shortest diffusion time in our study would be associated with distances larger than the typical sarcomere size, the observed differenced in AD are likely caused by other restrictions. Further research is warranted to elucidate the cause for the observed changes in AD upon muscle contraction.

The motion of actively contracting tissue is expected to be somewhat coherent, and our results clearly indicate that trapezoid-cosine OGSE waveforms, being motion compensated, are able to fully refocus spin dephasing due to coherent motion. While residual areas of signal voids are observed even when encoding diffusion using an OGSE approach, robust tensor fitting with outlier rejection offers robustness toward these artifacts and allows to obtain reliable diffusion estimation. While diffusion values for actively contracted skeletal muscle have not been previously reported, our AD and RD results obtained in relaxed muscle are in agreement with previously reported values obtained using conventional PGSE DTI in relaxed musculature (1). Mazzoli et al. performed a DTI experiment in relaxed calves using a PGSE approach and reported RD values of 1.55 ± 0.06 mm^2^/s for the tibialis anterior and 1.51 ± 0.04 mm^2^/s for the soleus and AD values of 2.32 ± 0.05 and 2.64 ± 0.07 mm^2^/s (4). Similarly, Schlaffke et al. (30) reported 1.89 ± 0.08 and 2.14 ± 0.22 mm^2^/s for AD and 1.35 ± 0.13 and 1.62 ± 0.21 mm^2^/s for RD, for the tibialis anterior and soleus, respectively. Therefore, our results indicate that OGSE could be used to obtain a reliable diffusivity assessment in the lower leg. We should point out that the limited gradient strength of clinical MRI systems, together with peripheral nerve stimulation (PNS) constraints, resulted in relatively low b-value and long TE, which are suboptimal for DTI measurements in the skeletal muscle. However, while the OGSE sequence might not be optimal for routine DTI measurements, it could be highly beneficial to study actively contracted muscles or to image subjects who cannot keep their musculature fully relaxed during the full duration of the DTI experiment, as shown by its robustness to (in)voluntary muscle contraction.

Diffusion in the skeletal muscle is conventionally measured using a PGSE monopolar diffusion scheme with moderately short gradients and large gradient amplitude (28), resulting in moderate diffusion times. This monopolar approach however is not suitable if one wants to investigate time-dependent diffusion in the short time regime, due to its inherent inability to achieve sufficient diffusion encoding at short diffusion times. On the other hand, OGSE can achieve higher diffusion sensitivity for short diffusion times. Additionally, even though short gradients are less sensitive to motion compared with the longer PGSE gradients used in this study, they are still highly susceptible to signal dephasing due to contraction (31) and, therefore, unsuitable to obtain DTI information during muscle active muscle contraction. Our study suggests that fully compensated PGSE waveforms (M_0_ = M_1_ = M_2_ = 0) could provide the same benefit as OGSE for skeletal muscle contraction imaging, although this hypothesis has to be tested. If time-dependent diffusion is not of interest, PGSE with first- and second-order motion compensation might provide a higher b-value than OGSE (32), which could be further improved by numerical optimization (33, 34).

DTI experiments using a monopolar PGSE waveform, which is not motion compensated, resulted in complete suppression of signal originating from flowing spins, as indicated by the dark blood vessels. On the other hand, when flow-compensated gradient waveforms, such as cosine OGSE, are used, coherent blood flow is no longer dephased, as indicated by the bright vessels in diffusion-encoded volumes, as previously observed (35). This could create an overestimation of diffusion values close to vessels due to partial volume effects when using compensated vs. uncompensated gradient waveforms. However, care was taken to avoid blood vessels during manual delineation of the tibialis anterior and soleus muscles, and our results show that RD and AD in resting musculature are higher for PGSE than for OGSE approaches even for non-contracting musculature. Therefore, partial volume effects with uncompensated blood flow are expected to have a limited effect on our results.

The measured AD and RD values did not show a clear time-dependent behavior in the soleus and tibialis anterior. Time-dependent diffusion in the cross section of skeletal muscle fibers has been investigated at longer diffusion times (up to 1 s) using stimulated echo approaches (13, 14), with lower diffusion coefficients measured for increasing diffusion times, likely indicating greater restriction effects of water molecules by cellular membranes. On the other hand, this study shows higher diffusivity values for PGSE, which has a longer diffusion time. Perfusion effects can lead to overestimation of diffusion coefficients (36), and the higher values of diffusivity observed in this study for the PGSE sequence compared with OGSE even in relaxed musculature could be explained by higher sensitivity to perfusion effects due to the lack of motion compensation (35). Therefore, based on this preliminary work, OGSE sequences with lower frequencies could be preferred, due to the possibility of achieving higher b-values for the same TE, which could further reduce the residual effect of perfusion on the measurements. The presence of a clear anisotropic diffusion behavior but no increase of diffusion values as a function of diffusion time (7.5 and 4.1 ms for OGSE 25 Hz and OGSE 50 Hz, respectively) could indicate a restricted diffusion behavior for all investigated sequences. This restricted diffusion behavior is likely not caused by the myofiber membranes, which are characterized by longer length scales but rather by ultrastructural restrictions. Further research is needed to elucidate the connection between the measured diffusion values and the underlying tissue structure.

This study has a number of limitations. First, this proof-of-concept study only included a limited number of subjects. Future research will focus on applying this method to study muscle contraction mechanisms in a larger number of healthy subjects and patients with neuromuscular diseases. Another potential limitation of our study is the relatively long TE of the acquisition, required to obtain a sufficient amount of diffusion encoding. This could have biased our analysis by increasing diffusion sensitivity to the extracellular space. Additionally, the limited gradient strength available on our clinical system, combined with compliance with PNS requirements, resulted in relatively low diffusion encoding strength, which might generate sensitivity to perfusion. Active muscle contraction could locally increase blood perfusion, which could lead to an overestimation of DTI parameters (36). Wu et al. (37) showed the dependency of diffusion contribution to the diffusion signal through the intravoxel incoherent motion (IVIM) model to be dependent on the average distance that blood can travel during the diffusion times, with smaller perfusion coefficients measured at shorter diffusion times. This work therefore suggests the potential of measurements at shorter diffusion times to minimize the effect of perfusion. Additionally, fully motion-compensated waveforms, such as the trapezoid-cosine waveforms used in this study, were also shown to minimize the influence of perfusion in the liver in Moulin et al. (35), but our result could still be partially biased by residual perfusion contributions. Future work will focus on combining OGSE diffusion encoding with additional orthogonal gradients, as described by Wu et al. (37) for brain applications, to completely eliminate the influence of residual perfusion on the quantitative diffusion results. This promising method, However, we should mention that this promising method was implemented on a preclinical system with much higher gradient strength than currently achievable on a clinical system and will therefore require adaptations for applications in human skeletal muscle DTI. Lastly, our experimental setup did not allow for controlled and standardized force production during muscle contraction. Future studies will investigate the effect of maximal voluntary contraction (MVC) on the diffusion results.

## Conclusions

In conclusion, this study demonstrates that OGSE diffusion encoding allows for quantitative DTI imaging of actively contracting human musculature. The use of cosine trapezoids diffusion encoding waveforms led to reduced signal voids in diffusion-weighted images, which could be used to calculate DTI parameters. Additionally, different diffusion behaviors were observed for relaxed muscle and musculature at different levels of contraction. Taken together, our results showed that OGSE holds the potential to non-invasively assess microstructural changes occurring in the skeletal muscle during contraction and for the non-invasive assessment of contraction abnormalities in patients with muscle disease. These results could lead to a deeper understanding of muscle contraction abnormalities and the optimization of treatment and intervention strategies.

## Data Availability Statement

The raw data supporting the conclusions of this article will be made available by the authors upon request, without undue reservation.

## Ethics Statement

The studies involving human participants were reviewed and approved by Stanford University Institutional Review Board. The patients/participants provided their written informed consent to participate in this study.

## Author Contributions

VM conceived, designed the study, and acquired the data. VM and KM analyzed and interpreted the data. VM drafted the manuscript. KM, FK, BH, and GG revised it. All authors gave their approval of the final submitted version.

## Conflict of Interest

The authors declare that the research was conducted in the absence of any commercial or financial relationships that could be construed as a potential conflict of interest.
